# Pilot evaluation of physical and psychological effects of a physical trek programme including a dog sledding expedition in children and teenagers with cancer

**DOI:** 10.3332/ecancer.2015.558

**Published:** 2015-07-28

**Authors:** Clothilde Vallet, Nicolas André, Jean-Claude Gentet, Arnauld Verschuur, Gérard Michel, Frédéric Sotteau, Cécile Martha, Laurent Grélot

**Affiliations:** 1 Association ‘Sourire à la Vie’, Marseille 13016, France; 2 Research Unit EA 3279 and Department of Public Health, Hôpital de la Timone, Marseille 13005, France; 3 Department of Paediatric Haematology and Oncology, Hôpital de la Timone Enfant, Marseille 13005, France; 4 Aix Marseille Université, Inserm, CRO2 UMR_S 911, Marseille 13005, France; 5 Aix-Marseille Université, CNRS, ISM UMR 7287, 13288 Marseille Cedex 09, France; 6 Faculty of Sport Sciences, Aix-Marseille University, Marseille 13009, France

**Keywords:** children, oncology, solid tumour, leukaemia, self-esteem, physical fitness, general physical preparation, health, sport

## Abstract

**Aim of the study:**

To evaluate the feasibility and to measure the effects of a six-week-long adapted physical activity programme (APAP), including 5 days of intense dog sledding, on the physical and psychological health of children and adolescents treated for cancer.

**Methods:**

Eleven children and teenagers (4 girls, 7 boys; mean age 14.3 ± 2.9 years) participated in this monocentric pilot programme of adapted physical activities from February 2013 to March 2013. Seven were still on treatment. The programme lasted 6 weeks. A series of physical tests and psychological questionnaires were carried out before and after the programme.

**Results:**

All children and teenagers completed the full programme. An improvement in all physical and psychological parameters was observed. Statistically significant differences were observed for global self-esteem (6.2 ± 2.1 to 7.7 ± 1.8; *p* = 0.02), perceived sport competence (5.3 ± 3.2 to 7.4 ± 2; *p* = 0.02) and perceived physical strength (5.6 ± 2.5 to 7.1 ± 1.8; *p* = 0.001). Regarding physical tests, the physical training led to statistically significant improvement for sit-ups (13.8 ± 2.6 to 21.75 ± 5.4; *p* = 0.01), muscle tone (76 ± 23.7 to 100 ± 22.9; *p* = 0.01), and resting heart rate (96.1 ± 3.2 to 91.6 ± 4.5; *p* = 0.03).

**Conclusion:**

This programme is feasible in children and adolescents even during their oncologic treatment. During the 6-week programme, children and adolescents improved their physical and psychological health, and the putative benefits of the APAP are discussed. A larger randomised trial started in 2014.

## Introduction

Cancer in children represents less than 1% of all cancers but is a real public health issue because it remains one of the main causes of mortality in younger people in high-income countries. Each year, 160,000 new cases are diagnosed with 90,000 deaths worldwide, and among these new cases, 15,000 are diagnosed in Europe [1].

Current therapeutic advances (new drugs and novel therapeutic strategies, new surgical techniques, and new radiation modalities) have led to the cure of about 80% of children and adolescents with cancer. Nevertheless, side effects and long-term sequelae, whether related to the disease or treatments, can lead to mild to severe handicaps affecting the health and daily life of children and adolescents during and after completion of the treatment [2–6]. Indeed, two-thirds of survivors will experience a late effect. Among them, a quarter is severe or lifethreatening [6]. Moreover, children and teenagers with cancer are immersed in a painful experience with an uncertain outcome and social life, relational, familial, and schooling disruptions.

Continuous decline in physical abilities, and fatigue [7] related to both long stays in hospital [8] and the use of cytotoxic drugs, is an important issue. Indeed, studies have demonstrated that cancer related treatments have a negative impact on both physical and psychological health in children [9–11], including a decrease in physical capacity, self-esteem, and an increase in anxiety state [12–13]. On the one hand, in adults, several studies have shown that exercise during or after treatment could lead to an improved physical condition and social life. Specifically, aerobic exercise is now strongly recognised as beneficial for individuals with cancer-related fatigue during and post-cancer therapy, in particular those with solid tumours [14]. On the other hand, there are few studies in the literature regarding this topic in relation to children [15–18]. Nevertheless, some studies have observed an increase in quality of life and a trend towards an improvement in fitness in children who perform physical exercise during or after treatment [19–23] and a decrease in fatigue [7].

This study aimed to evaluate the feasibility of an adapted physical activity programme (APAP) and the beneficial effects on the physical and psychological performance of children and teenagers suffering from cancer. Since the mid-1970s, the term ‘adapted physical activity’ (APA) has referred to physical activity interventions aiming to enable and enhance the performance and participation of people with disability. Entering as a specialty in the curriculum of French university studies in the faculties of Sport Sciences and Physical Education, APA is now recognised as a professional field of study and intervention with a strong cross-disciplinary knowledge base. In the team of ‘Sourire à la Vie’, CV and FS are certified professionals of APA under the direction and supervision of whom the adapted physical activities were conducted. Thus, our APAP was offered to children and teenagers during their treatment or within 1 year after the end of treatment. Unlike a few previous studies [24] investigating the putative benefits of intra-hospital conditioning programs on young cancer patients, one of the major novelties of our programme relies on its ‘ecological approach’. Indeed, children and teenagers were engaged in activities outside of the hospital setting, over several weeks including the period of treatment (chemotherapy, surgery, and radiotherapy) aiming to prepare for their participation in an expedition with sled dogs in Canada organised by Sourire à la Vie. Founded in 2006 by FS and LG, Sourire à la Vie is a non-profit organisation law 1901 (http://www.sourirealavie.fr/) that takes care of young people with cancer by promoting and organising adapted (soft) physical activities outside the hospital. These activities, performed throughout the year under the supervision of certified professionals in APA, include dance, cruising sail boarding, trekking, and snow activities such as adaptive skiing, snowshoeing and dog sledding.

Hence, this study might be viewed as an opportunity to quantify the putative health benefits and well-being commonly observed in young patients after a stay at Sourire à la Vie by the healthcare workers (nurses, oncologists, and psychologists) of the Department of Haematology and Oncology of La Timone Children Hospital. In contrast, due to the heavily constrained context, investigations routinely performed in the field of physical activity sciences (e.g., VO_2_ measurements to quantify exercise intensities) simply could not be done here, thus limiting the extrapolation of the present results to other groups of patients.

## Materials and Methods

### Patients

All children and adolescents were treated in the Department of Paediatric Haematology and Oncology of La Timone Children Hospital AP-HM, France. Moreover, there were active members of Sourire à la Vie. Patients with any type of paediatric malignancies were selected after validation by the medical staff. The selected patients needed to fulfil the following two criteria: medical capability to participate (i.e., according to the timing of treatment and medical evaluation, fitness) and willingness to participate to the programme (both the stay in Canada and the physical and psychological preparation and testing associated with this study). Young patients and legal guardians gave written consent for participating. The study has been approved by the local ethics committee. Patients were treated according to the European or national protocols corresponding to their underlying malignancy.

**Figure 1.**
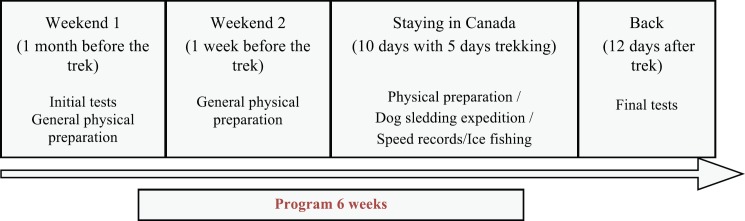
Details of the programme and evaluation procedures. Although mentioned in the figure, initial and final testing are not part of the programme per se.

The APAP, developed by the Association Sourire à la Vie, lasted 6 weeks and is described in Figure 1. Participants joined for two weekends of two days of preparation, outside of the hospital before of the joining the expedition.

Background information (demographics, underlying malignancies) were obtained from clinical charts.

The physical and psychological health of children and adolescents was evaluated on the first and on the last day of the programme (Figure 1). The programme began with physical and psychological tests presented below and assessed by a single investigator/observer from the association (CV) just followed by a physically active session. The children and adolescents were further evaluated 12 days after the completion of the expedition to limit as much as possible the potential negative effects of jet lag and were asked to give feedback on their fitness. For the physical evaluations, we selected very simple tests so that the children and adolescents required only a few minutes or tries for familiarisation with the equipment and testing procedures. Moreover, this limits the possibility of a learning effect between the initial and the final sessions of testing.

We evaluated aerobic fitness with the six-minute walking test (6MWT). The test involves walking on a flat surface of at least 20 metres and a round trip. The objective was to walk the greatest possible distance in 6 minutes. This is one of the most widely used submaximal tests to assess cardiovascular endurance in several healthy or weakened populations. This test is well tolerated by children and is very suitable for the evaluation of the functional status of people for whom a maximal test is contraindicated or impractical [25–28]. For this test, heart rate (HR) was evaluated in the children and adolescents with an HR monitor (FT1 Polar©, Finland) at rest (resting HR), during exercise (HR maximal effort) and recovery. These cardiac frequencies allowed calculation of a recovery factor, and the value for the recovery of HR was defined as the reduction in the HR from the rate at peak exercise to the rate 3 minutes after the cessation of exercise.

All the tests used for this study are part of the validated EUROFIT battery [29]. This European battery of cardiovascular and motor tests was first designed for assessing school-aged children’s physical development. Its easiness of use makes it accessible while respecting the requirements of validity, accuracy, and objectivity [30]. Moreover, the Eurofit test battery offers a wide range of reference data across ages, countries, and diseases [31].

The battery of physical fitness tests included the assessment of the following physical fitness measures:

1. Abdominal muscle endurance and strength was measured by the number of correctly completed sit-ups in 30s. Sit-ups were performed with the hands placed at the side of the head, knees bent at 90°, and the feet secured by the investigator. A full sit-up is defined as touching the knees with the elbows and returning the shoulders to the ground.
2. Functional postural muscle structure was measured by a test of ‘muscle tone’, a maximum time held in the following position: elongated front support position on forearms with right angles and the tips of the toes, horizontal trunk (closed and parallel legs).
3. Evaluation of strength of the upper limb was performed by measuring the distance at which a medicine ball (Decathlon, France) was thrown. The weight of the ball depended on children’s weight (1 or 2 kg for bellow or above 12 years old, respectively).
4. Evaluation of strength of the lower limbs was performed using a Myotest Sport© measurement tool (Switzerland). We evaluated the standing vertical jump during a countermovement jump: the young patient started from an upright standing position, made a downward movement by flexing at the knees and hips, then immediately extended the knees and hips again to jump vertically up off the ground; he repeated the stroke 5 times (measurement of flight height (centimetres), power (watts per kilogram), strength (newton per kilogram) and jump speed (centimetres per second).

Psychological health, specifically self-esteem and physical self-perception, was measured using the physical self-inventory (PSI-6, [32]). This shortened version of the original PSI (25 items) is based on a single-item self-assessment related to six dimensions of self-perception: global self-esteem/physical self-worth/physical condition/sport competence/physical attractiveness/physical strength, with a horizontal visual analogue scale (VAS) of 10 cm, from 0.0 to 10.0 absolutely. Although the PSI-6 has not been validated in paediatric oncology, it was appropriate for the purpose of our study since it is a very brief tool, which contributes to the reduction in fatigue, frustration, and boredom associated with answering highly similar items repeatedly. Moreover, it has been validated in the French language [32] and has been used in studies investigating physical self-concept among French adolescents [33].

## Adapted physical activity programme

The general physical preparation (GPP) was conducted in a fun, child-friendly atmosphere. Due to the putative weak physical fitness of the children and adolescents undergoing treatment, GPP consisted here solely of general conditioning to improve strength, speed, endurance, flexibility, skill, and the body’s ability to handle greater workloads (i.e., decreasing fatigability). GPP was conducted by APA instructors in the same way as it is carried out in physical education classes in a school setting. Typically, the period of physical activities lasted from 60 to 120 minutes, and they were scheduled 1 to 5 times a week (details given below). The intensity of physical activity was adjusted to each young patient according to their age and level of fitness. In addition, on-going adjustments were performed during the sessions according to both feedback of the young patients (i.e., perceived level of physical exertion) and the objective capability to follow the instructions of the APA professionals.

To assess the effectiveness of our GPP, we used a battery of tests dealing with endurance, resistance, strength, flexibility, and balance workout. The sessions contained various methods and materials and the intensity, number of repeats and recovery time for exercise were individually adapted to each patient. The general physical preparation (GPP) was conducted as follows.

1. Warm-up exercises: Joint rotation or automassage muscle, to warm-up the muscles (with or without ball) (10 minutes)/cardio warm-up: trots and little fun games (20–30 minutes)/active stretching (5 minutes);
2. Muscle building (30 minutes): Circuit between different fun workshops for working the lower limbs, upper limbs, and trunk (abdominal muscles, muscle tone, push-ups, small dumbbells, plates of balance and proprioception, gym ball, medicine ball);
3. Aerobic fitness (30 minutes): Treadmill (i.e., simulation of the up and down sledding at different speeds), exercise bike, running in the courtyard with stairs, jump rope;
4. Recovery (15 minutes): Stretching and breath work.

An additional boxing session (60 minutes including recovery periods) was also conducted in order to work on the fighting spirit and a session of physical expression (dance, physical expression; 120 minutes with recovery periods) to work on trust, cooperation and communication was also performed. Advice on stretching, injury prevention, body aches, nutrition, hydration, and sleep was also provided to the children.

This GPP programme was followed by a 5-day long expedition in a camp in the forest in Quebec (Canada) and nearby lakes. A session of physical training in the snow and a first contact with the pack of dogs and the sleds were held the day before departure. From day 1 to 5, between 2 to 5 hours per day of dog sledding sessions were set, including speed records. Moreover, the patients participated in sporting games in the snow and ice fishing on the frozen lakes. Globally, children practiced between 4 to 5 hours of physical activity per day, for a total of a minimum of 35 hours of activity throughout the programme (preparation + trek). All throughout the physical activity programme (PAP), sports and medical professionals accompanied participants.

## Statistical analysis

Commercial software (Statisca, Statsoft, Dell©) was used for all statistical analyses. The differences between evaluation performed at different time points were assessed using either the parametric test (*t*-test) when the data followed the normal distribution either by the non-parametric Wilcoxon test. Differences were considered statistically significant when *p* value was below 0.05.

## Results

We aimed at including 11 patients in the group travelling to Canada. Only two children refused to participate. Eleven children and adolescents participated in this programme. There were 4 girls and 7 boys. The mean age of participants was 14.3 ± 2.9 years. Among the 11 patients, seven were still in treatment and four were in remission and off treatment for less than one year. The mean value anthropometric parameters observed during the study were as follows: weight (48.4 ± 8.5 kg), height (154 ± 11 cm), and body mass index (BMI) (20.3 ± 3.0 kg.m^−2^). Details of some characteristics of the enrolled population are reported in Table 1.

All children completed the whole programme and were assessed on physical and psychological dimension of health, as detailed in Table 2.

Following the programme, PSI scores increased and the differences were significant for dimensions global self-esteem (6.2 ± 2.1 to 7.7 ± 1.8; *p* = 0.02), perceived sport competence (5.3 ± 3.2 to 7.4 ± 2; *p* = 0.02), and perceived physical strength (5.6 ± 2.5 to 7.1 ± 1.8; *p* = 0.001). The physical self-worth dimension tended to statistical significance (5.4 ± 2.3 to 7 ± 2.2; *p* = 0.06). For physical tests, the differences were significant for sit-ups (13.8 ± 2.6 to 21.75 ± 5.4; *p* = 0.01), muscle tone (76 s ± 23.7 s to 100 s ± 22.9 s; *p* = 0.01) and resting HR (96.1 bpm ± 3.2 bpm to 91.6 bpm ± 4.5 bpm; *p* = 0.03). The values of the distance in the 6MWT (602.3 m ± 36.9 m to 618.1 m ± 46.5 m; *p* = 0.06) and the strength of the upper limbs (3.41 m ± 0.32 m to 3.55 m ± 0.44 m; *p* = 0.09) also tended towards but did not reach statistical significance.

No injuries were reported during the participation of the children in the programme.

## Discussion

Given the relative lack of knowledge about the impact of ‘physical activity and sport’ on the health of children with cancer [15, 16, 24], we report here on the preliminary evaluation of an adapted physical activity program (APAP) performed outside of the hospital including a dog sledding expedition. The purpose of this study was to measure changes in physical and psychological health-related parameters of children and adolescents treated for cancer, whatever the time of treatment (initial phase of treatment, relapse, palliative care) during a 6-week physical activity programme. Thus, we report that an adapted physical program is feasible and harmless for children even during treatment and even in a palliative setting outside of the hospital. This may be at least in part related to the organisation of the programme that aimed at generating gratification through physical activities without concern for performance or evaluation, where fun/games are intended to mask the physical effort.

The APAP led to a statistically significant improvement of several physical parameters (muscle tone, sit-up, and baseline HR) and psychological issues (global self-esteem, sport competence, and physical strength). It should be noted that the 12-day delay between the end of the trek in Canada and the final testing session in France, chosen to remove the putative negative effects of a jet lag fatigue, might have slightly decreased the performance of the young patients by a weak deconditioning. Thus, physical parameters that depicted a non-statistical trend towards improvement (i.e., total distance in 6MWT, number of sit-ups, muscle tone, and upper limb strength) should be viewed with that time constraint. The results of physical tests support the results of PSI survey, which highlights the perception that children and adolescents have on the body image and self-esteem in general. Interestingly, in addition to having a mean 16 meters increase in the 6MWT, the heart recovery rate was maintained and the endurance test results showed improvement. Adaptation to the effort tends to be better since the coefficient of recovery was stronger in the final testing. This demonstrates the potential benefits on physical and psychological health of APAP such as the one we set. In addition, the 6MWT and measures of the strength of upper and lower limbs showed a trend towards improvement between the beginning and the end of the APAP, but without reaching a level of statistical significance. To the best of our knowledge, these observations have never been reported previously.

Among the items that were improved by the program (abdominal muscle endurance, sit-ups scores, muscle tone, baseline HR, self-esteem, sport competence, and physical strength, for psychological health), some had already been evaluated in children in a normal population, or in children with chronic diseases such as cancer. [20, 27, 34]. Thus, in a normal population, an average of 25.1 sit-ups was completed in 30 seconds by children with age ranging from 9 to 19 years (mean age 14 years). Our reported population scored well below these references at the beginning of program scores. During the initial testing, an average of 13.8 sit-ups was reached. However, the final test results are close to the scores of the normal population averaging 21.7 (i.e., a 57% increase in the number of sit-ups) strongly suggesting that such a programme is beneficial for maintaining abdominal muscle endurance of young cancer patients. Keats *et al* [7] reported similar improvements in their study, following a 16-week programme in children with cancer.

Our results for the endurance test are not statistically significant, although there was a trend towards an improvement of the distance reached after our programme. Noteworthy, this improvement may be in part related to a better adaptation to efforts as evidenced by the increase in the cardiac recovery factor. Of note, this indicator may be taken with caution as cardiac frequency greatly varies according to age.

Takken *et al* [27] compared the performance obtained with the 6MWT in children with end-stage renal disease (ESRD) and populations of children suffering from chronic diseases. Regardless of the type of chronic disease (end-stage renal disease, juvenile idiopathic arthritis, haemophilia, and spina bifida) results ranged from 60 to 90% lower than the reference values in normal population reported by Li *et al* [28] and Geiger *et al* [25]. Therefore, our results are consistent with these observations since the average distance measured with the 6MWT for our 11 young patients was also lower than those obtained in a healthy population [25, 28] (Table 3). Although some motor performances were improved during the 6-week programme, they mainly remained low in our child and adolescent patients. Thus, as others have done previously, we recommend that individualised exercise interventions to attenuate motor deficits and promote physical activity are performed during cancer treatment in order to enhance motor performance and improve social participation during and after cancer therapy [35].

Finally, our results for self-esteem questionnaires are consistent with other studies that have shown that quality of life is improved in patients with cancer following an exercise programme during their treatment. Thus, in children with cancer Speyer *et al* [20] reported a better quality of life during an adapted physical activity (APA) practice during periods of hospitalisation as compared to periods when children do not participate to the APA. The improvement mainly concerned self-esteem, physical capacities, and mental health.

Our pilot experience display several limits. First, it lacks statistical power because of the limited number of patients included in this programme. Then, children and adolescents were also selected based on their ability to perform the entire programme, including the ability to perform the trek in Canada, likely generating a bias by selecting the fittest ones. Thirdly, we only evaluated the short-term impact of the programme since evaluation was performed only 12 days after the trek. Longer follow-up is necessary to evaluate whether the observed benefits are sustained or not. In addition, while the duration of each period of physical activity was individually quantified for each child and adolescent throughout the APAP, the exact intensity (i.e., light, moderate or heavy) of each type of activity was missing. Lastly, it’s difficult to compare our results to other paediatric studies since they were mainly performed in children with acute lymphoblastic leukaemia and because of an important heterogeneity in the modalities of training (type, duration, frequency, and intensity). Finally, the study lacked an age- and gender-matched control group (healthy children and adolescents), but due to both hospital and sojourn in Canada (‘ecological’) contexts, this was clearly impossible to achieve.

## Conclusion

The practice of physical activity is essential in the development of a child [36–37], and the diagnosis of cancer should not deprive the child of physical activity. APAP should, *a priori*, have a place in the care of children with cancer. We report here on the evaluation of a pilot physical training programme in children and teenagers treated for cancer. All participants successfully completed the programme without harm. We observed statistically significant beneficial effects on both physical and psychological health between the initial and the final testing sessions of the programme. However, these results need to be confirmed and generalised by a state-of-the-art randomised study with a longer training programme, including a larger number of patients in order to make meaningful conclusions. This pilot study shows the harmless feasibility of such an APAP with children and adolescents undergoing cancer treatment.

## Figures and Tables

**Table 1.** Population’s characteristics.

**Age**	14.3 ± 2.9 years
**Height**	154 ±11 cm
**Weight**	48.4 ± 8.5 kg
**BMI**	20.3 ± 3.0 kgm^−2^
**Sex** Girls Boys	4(36.4%) 7(63.6)
**Type of cancer** Leukaemia (1 ALL, 1 AML) Rhabdomyosarcoma Non-Hodgkin’s lymphoma Brain tumour (low-grade glioma) Langerhans cell histiocytosis Wiedemann–Beckwith syndrome	2(18.2%) 3(27.3%) 2(18.2) 2(18.2) 1(9.1%) 1(9.1%)
**Stage treatments** Receiving treatment Palliative care Off treatment in remission	4(36.4%) 3(27.3%) 4(36.4%)

BMI: Body Mass Index; ALL: Acute Lymphoblastic Leukaemia

**Table 2.** Evolution parameters assessing the physical and psychological dimensions of the health of children and adolescents between the beginning (pre-test) and end (post-test) of the 6 weeks AP programme.

	Pre-test (M ± SD)	Post-test (M ± SD)	*p* value
**Physical parameters**			
**Endurance** Total distance (6MWT) (m) Resting HR (bpm) HR max effort (bpm) Recovery factor (bpm)	602.3 ± 36.9 96.1 ± 3.2 185.3 ± 17.2 57 ± 11.2	618.1 ± 46 91.6 ± 4.5 184.6 ± 18.1 60.5 ± 7.8	0.06 0.03 0.88 0.61
**Sit-ups(nb/30s)** **Muscle tone (s)** **UL strength (m)**	13.8 ± 2.6 76 ± 23.7 3.41 ± 0.32	21.75 ± 5.4 100 ± 22.9 3.55 ± 0.44	0.011 0.011 0.09
**Strength LL** Jump height (cm) Might (W/kg) Strength (N/kg) Speed (cm/s)	18.2 ± 7.1 29.9 ± 8.7 19.8 ± 2.9 184.8 ± 38.2	20.2 ± 5.2 30.5 ± 7.3 20.8 ± 3.6 197.1 ± 26.2	0.68 0.75 0.46 0.7
**Pshycological parameters PSI (score)** GSE PSW PC SC PA PS	6.2 ± 2.1 5.4 ± 2.3 6.2 ± 2.3 5.3 ± 3.2 5.8 ± 2.1 5.6 ± 2.5	7.7 ± 1.8 7 ± 2.2 7 ± 2.2 7.4 ± 2 6.4 ± 2 7.1 ± 1.8	0.02 0.06 0.17 0.02 0.21 0.001

SD: Standard Deviation; HR: Heart Rate; LL Strength: Strength of lower limbs

UL Strength: Upper limb strength; PSI: Physical Self-inventory; GSE: Global Self-esteem

PSW: Physical Self-worth; PC: Perceived Physical Condition; SC: Perceived Sport Competence

PA: Physical Attractiveness; PS: Perceived Physical Strength; *p*: Level of Significance (statistical test pre versus post)

**Table 3.** Results of six-minute walking test.

6MWT	M ± SD
6MWT – Initial distance (m)	602.3 ± 36.9
6MWT – Final distance (m)	618.1 ± 46.5
Predicted 6MWD – Li (m)	666.8 ± 30.9
Predicted 6MWD – Geiger (m)	663.2 ± 37.7
% Predicted – Li Initial Final	90.3% 92.7%
% Predicted – Geiger Initial Final	90.8% 93.2%
